# Etonogestrel implant for LH suppression in a novel DuoStim ovarian stimulation protocol using overlapping doses of corifollitropin alfa for social egg freezing: a case report

**DOI:** 10.5935/1518-0557.20250033

**Published:** 2025

**Authors:** Bruno Ramalho de Carvalho

**Affiliations:** 1 Bruno Ramalho Reprodução Humana, Centro Universitário de Brasília - CEUB, Brasília, DF, Brazil

**Keywords:** ovarian stimulation, fertility preservation, etonogestrel implant, progestin-primed ovarian stimulation, corifollitropin alfa

## Abstract

This article reports the case of a healthy nulliparous woman, 34 years old, weighing 70 kg with a BMI of 23.67 kg/m^2^, who was using an ENG 68 mg implant for contraception and was experiencing amenorrhea. She presented with 19 antral follicles on initial ultrasound (US) and desired social fertility preservation. Follicular phase controlled ovarian stimulation (COS) was initiated with the administration of corifollitropin alfa (CFA) 150µg on day 1, followed by an overlapping dose of CFA 100µg on day 5. US monitoring began on the ninth day of stimulation, and a trigger shot with recombinant chorionic gonadotropin (rhCG) 250µg was administered on day 11 when six follicles measured ≥16 mm. Follicular aspiration (FA) occurred 36 hours later, resulting in the retrieval of 13 oocytes; 11 were metaphase II (MII) and were vitrified. Luteal phase COS began one day after the first FA, using CFA 150µg. The trigger was administered again on day 11, with rhCG 250 µg, when eight follicles were ≥16 mm, followed by FA 36 hours later, resulting in the retrieval of six MII oocytes, all of which were vitrified. Our report is the first to highlight that a woman attempting fertility preservation while using an ENG implant for contraception may benefit from LH suppression during COS. Additionally, it proposes a novel protocol that applies CFA in overlapping doses, aiming for comfort and lower cost.

## INTRODUCTION

Patient-friendly controlled ovarian stimulation (COS) protocols for medically assisted reproduction (MAR) have been studied over the last two decades, focusing on reducing costs, physical and emotional burdens, and risks while maintaining access equity without compromising efficacy (Pennings & Ombelet, 2007; Devroey *et al.*, 2009). Indeed, improving the experience during COS must be a priority for the assistant physician, as more than half of the women attempting oocyte pickup feel that the treatment negatively impacts their daily lives and are concerned about potential errors in gonadotropin administration (Huisman *et al.*, 2009).

Corifollitropin alfa (CFA), a long-acting follicle-stimulating hormone (FSH), allows a single subcutaneous dose to replace seven daily injections. This aligns well with the principles of a patient-friendly COS, as it may provide women with comfort and reproductive outcomes similar to those observed with other gonadotropins (Pouwer *et al.*, 2015; Cozzolino *et al.*, 2019; Budani *et al.*, 2023).

Additionally, in alignment with patient-friendly COS precepts, oral progestins have been utilized to suppress luteinizing hormone (LH) surge in MAR, as a substitute for subcutaneous gonadotropin-releasing hormone analogues (GnRHa), exhibiting similar rates of precocious follicular rupture and treatment outcomes (Cui *et al*., 2021; Ata & Kalafat, 2024).

Assuming that progestin-induced LH suppression occurs by any route of administration and that overlapping CFA doses may ensure sustained follicular stimulation until the maturation trigger criterion is reached, we report the case of a patient attempting social egg freezing in a DuoStim protocol with overlapping CFA doses, utilizing the etonogestrel (ENG) subdermal implant-used as a contraceptive-for LH suppression.

## CASE REPORT

The patient was a 34-year-old healthy single nulliparous woman attempting egg freezing for social fertility preservation. She reported using the ENG 68 mg implant (Implanon, N.V. Organon, Oss, Netherlands) for contraception, which was placed twelve months prior, and she was in amenorrhea. At her first appointment, she weighed 70 kg and had a body mass index of 23.67 kg/m^2^. Her physical examination was uneventful. The patient’s medical history included a previous laparoscopic exploration of a ruptured ovarian cyst without the need for oophoroplasty.

COS began on an arbitrary day, as per the patient’s request, following the identification of 19 antral follicles in quiescent ovaries through the administration of corifollitropin alfa (CFA) 150 µg (Elonva®, NV Organon Oss, Netherlands). On day 5, an additional dose of CFA (100 µg, Elonva, NV Organon Oss, Netherlands) was administered. Follicular development was monitored via transvaginal ultrasound scans on stimulation days 9 and 11, at which point six follicles measured ≥ 16 mm and recombinant chorionic gonadotropin (rhCG) 250µg (Ovidrel®, Merck Serono S.p.A., Bari, Italy) was administered to trigger maturation. Follicular aspiration (FA) took place 36 hours later. The aspiration of the 14 larger follicles led to the retrieval of 13 oocytes, of which 11 were in metaphase II (MII) and were subsequently vitrified. To facilitate a second oocyte retrieval following a new gonadotropic stimulation cycle in the luteal phase, eleven small follicles were left intact during the punctures.

Luteal phase COS was initiated following FA, with a new administration of CFA 150 µg (Elonva®, NV Organon, Oss, Netherlands). Transvaginal ultrasound scans monitored follicular development on stimulation days 5 and 9, and the trigger using rhCG 250µg (Ovidrel®, Merck Serono S.p.A., Bari, Italy) was scheduled for stimulation day 11, when eight follicles were considered to be ≥ 16mm. FA was performed 36 hours later. Aspiration of 15 large and small follicles resulted in the retrieval of six oocytes, all of which were in metaphase II (MII) and were vitrified.

**Figure 1:**
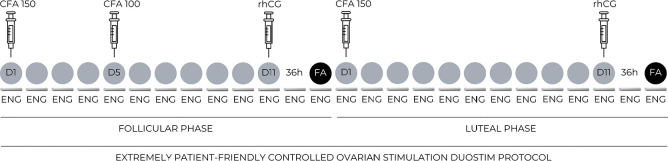
Diagram of a patient-friendly controlled ovarian stimulation protocol, utilizing overlapped doses of corifollitropin alfa (CFA) and leveraging a contraceptive etonogestrel (ENG) implant for LH surge suppression. D, day; rhCG, recombinant human chorionic gonadotropin; FA, follicular aspiration.

The DuoStim protocol (Figure 1) was well tolerated, and the patient reported only pelvic cramps as complaints, which resolved with oral antispasmodics during the first days of luteal phase stimulation.

## DISCUSSION

This is the first report of a patient undergoing COS and utilizing a contraceptive ENG implant for LH suppression. Along with the absence of follicular rupture, an adequate response was documented, leading to the vitrification of 17 MII oocytes from three overlapping doses of CFA administered in a very comfortable DuoStim protocol (150µg on stimulation day 1 plus 100µg on stimulation day 5 of the follicular phase, and a single dose of 150µg on stimulation day 1 of the luteal phase).

Despite the pursuit of new COS strategies in recent years, alleviating the physical and emotional burden of MAR remains a challenge. Established protocols with proven efficacy may offer a significant improvement. The psychological and physical stresses of COS are the main reasons women discontinue treatment after their first in vitro fertilization (IVF) cycle. Financial concerns also rank among the motivators (Verberg et al., 2008; Troude *et al*., 2014). This may explain why only 39% of women return for a second attempt (Grynberg *et al.*, 2023).

To reduce the burden of sequential self-administered gonadotropin injections, the comfort provided by the sustained release of CFA, which allows the patient to replace seven injections with one, was initially validated by 75% of oocyte donors, suggesting an increase in treatment compliance (Requena *et al.*, 2013). This is considered a significant quality since the reproductive outcomes of CFA are similar to those observed for daily gonadotropins, with statistical superiority in the total number of retrieved oocytes (Pouwer *et al.*, 2015; Budani *et al.*, 2023).

Overlapping doses of corifollitropin alfa are expected to provide patients undergoing follicular stimulation with fewer injections, contributing to a more comfortable experience compared to multiple daily subcutaneous injections of gonadotropin. In the protocol we propose, the overlapping dose is administered on day 5, a time when the circulating CFA concentration is approximately midway between the maximum concentration reached and the therapeutic threshold, according to early studies on its pharmacokinetics and pharmacodynamics (Duijkers *et al.*, 2002). It is important to clarify that the intention of administering a minor second dose of CFA was to prevent gonadotropin excess since it would overlap with the first dose.

Additionally, several oral progestins have been tested for LH suppression in MAR, resulting in very low rates of premature LH surge, a comparable number of competent oocytes and embryos, reduced rates of ovarian hyperstimulation syndrome, and similar embryo ploidy and pregnancy rates when assessed against conventional stimulation protocols (La Marca *et al.*, 2020; Ata *et al.*, 2021; Cui *et al.*, 2021; Lin *et al.*, 2023; Wang *et al.*, 2023). However, while oral administration itself may be viewed as an advantage compared to injections, the possibility of incorporating an in-situ ENG contraceptive implant into the COS protocol certainly aligns with enhancing patient-friendliness.

An AFC of 19 might raise concerns when considering the risk of ovarian hyperstimulation syndrome. However, reviews of CFA trials showed that women were at risk of moderate to severe or severe OHSS only when the threshold of 19 follicles ≥11mm was reached on the day of hCG (Griesinger *et al.*, 2016).

It is also important to note that recent data are reassuring regarding the use of PPOS as a safe and effective alternative for high responders (Xiao *et al*., 2019; Guan *et al.*, 2021; Huang *et al.*, 2021; Zhu *et al.,* 2021). In a recent meta-analysis, PPOS was correlated with a lower risk of OHSS compared to the GnRH-antagonist protocol (Deng *et al.*, 2024).

Regarding DuoStim, we agree with Garcia-Velasco *et al.* (2023) that the method may reduce treatment dropout and is potentially cost-effective. Additionally, we argue that DuoStim may be considered an interesting protocol when a cohort of new follicles develops during ovarian stimulation but does not reach trigger criteria in synchronization with the leading peers; this is why DuoStim was proposed in the case presented in this paper.

Finally, to the best of our knowledge, this novel protocol using overlapping doses of CFA was first presented by the authors of this article as a case report in 2023 during the XXVII Meeting of the Brazilian Society for Assisted Reproduction in Aracaju/SE, Brazil (Fonseca *et al.*, 2024), followed by its publication (Fonseca *et al.*, 2023). A recently published proof-of-concept study combining overlapping CFA doses and DuoStim showed promising results in a group of 15 patients (Castillo *et al.,* 2024).

To further enhance patient-friendliness, we believe that women and couples seeking the procedure should be involved in choosing the protocol, allowing them to consider factors such as comfort, affordability, and efficacy. The option to associate an already used contraceptive implant as part of the COS protocol, along with reducing the number of injections from overlapping CFA doses, could be an innovative approach in MAR. Of course, well-designed prospective studies are necessary to confirm the efficacy of this highly patient-friendly intervention.
